# Effectiveness of canakinumab treatment in Schnitzler's syndrome: a multi-center randomized placebo-controlled study

**DOI:** 10.1186/1546-0096-13-S1-O66

**Published:** 2015-09-28

**Authors:** K Krause, A Tsianakas, N Wagner, J Fischer, K Weller, M Metz, M Maurer

**Affiliations:** 1 Charité-Universitätsmedizin Berlin, Dermatology, Berlin, Germany; 2 Universitätsklinikum Münster, Dermatology, Münster, Germany; 3 Klinikum Darmstadt, Dermatology, Darmstadt, Germany; 4 Universitätsklinik Tübingen, Dermatology, Tübingen, Germany

## Background

Schnitzler's syndrome (SchS) is an adult-onset autoinflammatory disease characterized by urticarial exanthema and monoclonal gammopathy in combination with episodes of fever, arthralgia, fatigue, and bone and muscle pain. Anti-IL-1 targeting therapies in small patient numbers showed to be effective in reducing the clinical symptoms of SchS.

## Methods

The current placebo-controlled multi-center study was designed to assess the effects of the anti-IL-1ß monoclonal antibody canakinumab (CAN) on the clinical signs and symptoms of SchS. We randomly assigned 20 patients with active disease to receive CAN 150 mg or placebo s.c. injections (day 0). Following the evaluation of treatment responses on day 7 the study was continued by a 16-week open label phase with CAN injections upon confirmed relapse of clinical symptoms. Efficacy was determined by changes in the physician's global assessment (PGA; range 0-20), a combined symptom score which includes 5 key symptoms of SchS (urticarial rash, fever, fatigue, myalgia and arthralgia/bone pain), measurement of the inflammation markers C-reactive protein (CRP) and serum amyloid A (SAA) as well as changes in quality of life assessment (DLQI, SF-36).

## Results

CAN was highly effective in reducing median PGA total scores (14.0 to 2.0) as compared to placebo treatment (15.0 to 13.0) within 7 days after first administration (changes between treatment groups p < 0.0001). Median CRP reduced from 9.3mg/dL at baseline to 0.6mg/dL at day 7 in the CAN group vs. increase from 3.0mg/dL to 5.0mg/dL for the placebo group. Similarly, median SAA levels reduced from 428mg/L to 13mg/L for the CAN group vs. increase from 160mg/L to 205mg/L for the placebo group. The median changes from baseline to day 7 between treatment groups for CRP (p = 0.002) and SAA (p = 0.032) were significant. In addition, quality of life markedly improved. Changes in both physical component SF-36 scores and in DLQI sum scores were significantly greater (p < 0.0001) in the CAN vs. placebo group. The clinical and laboratory improvements were maintained during the open label phase of the study. Also, all placebo-treated patients responded well to CAN therapy during the open-label phase. Adverse events were manageable and included respiratory tract infections, gastrointestinal symptoms and hypertension.

## Conclusion

In this placebo-controlled study, CAN s.c. injections significantly improved the clinical signs and symptoms of SchS, reduced inflammation markers, and enhanced quality of life. CAN treatment may be considered a promising therapeutic option in these patients.

